# Neglected flexor tendon injury for 10 years, un-usual finding and one stage repair without free tendon graft: Case report

**DOI:** 10.1016/j.ijscr.2021.105902

**Published:** 2021-04-27

**Authors:** Khaled H. Mosallam

**Affiliations:** Hand and Reconstructive Microsurgery, Department of Orthopedic Surgery, Qena Faculty of Medicine, South Valley University, Egypt

**Keywords:** Flexor tendon, Tendon graft, FDS, FDP, Paneva-Holevich, Case report

## Abstract

**Introduction:**

In delayed or neglected cases of flexor tendon injury, reconstruction of flexor digitorum profundus (FDP) is usually performed using free tendon graft due to the retraction of tendon ends and shortening of the tendon. Flexor digitorum superficialis (FDS), palmaris longus or plantaris tendons can be used as a free tendon graft [1–3].

**Presentation of case:**

This is a case report of female patient 17 years old with neglected cut of her left Ring finger's FDP and FDS tendons zone II with suspected concomitant digital nerve injury, the injury was neglected for 10 years in the patient's non-dominant hand.

**Discussion:**

Upon exploration unusual finding of spontaneous healing of the proximal stumps of FDS and FDP tendons raised the idea of doing the repair one stage without free graft by using pedicled intra-synovial graft from the sublimis tendon to reconstruct the FDP tendon.

The patient after 4 months follow-up and after completion of the physiotherapy program regained the ability to actively flex her finger to near full flexion with improved function and cosmesis.

**Conclusion:**

Delayed flexor tendon reconstruction in neglected cases is still offering good results even after long periods of delay provided that the finger's joints are still supple and mobile.

## Highlights

• In neglected cases of flexor tendon injury, reconstruction of FDP tendon is usually performed using free tendon graft.
• The presence of two repair sites with free graft in between carries the risk of rupture or adhesion formation around the tendon graft.
• In this case report we succeed in doing the repair one stage without free graft.We used pedicled intra-synovial graft from the sublimis tendon to reconstruct the FDP tendon.

## 1 Introduction

This work has been reported in line with the SCARE 2020 criteria [4,5].

Primary repair of cut flexor tendons is indicated for the best results but secondary tendon reconstruction is still offering good results in the hands of experienced hand surgeons after following standard protocols [3].

Primary repair can be done either directly or using tendon graft [1].

Secondary reconstruction by tendon graft whether staged or not is usually needed if more than three weeks passed after injury due to the retraction of tendon ends and shortening of the tendon [1,3].

Staged tendon reconstruction is usually preferred in case of previously failed repair, if the pulleys are scarred or needs reconstruction, if the fingers joints are stiff or in the presence of bad skin or soft tissue [2,6,7,8].

The presence of two repair sites and free graft in between carries the risk of rupture of the graft ends or adhesion formation around the tendon graft [2].

## 2 Case report

Female patient 17 years old with neglected cut of her left Ring finger's FDP and FDS tendons zone II with suspected concomitant digital nerve injury, the injury was neglected for 10 years in the patient's non-dominant hand.

Pre-operative examination showed that the finger could be passively flexed with supple joints and pliable skin.

The main complaint of the patient was cosmetic, when she became a teenager, she disliked her hand appearance due to the extended position of her middle finger while flexing other fingers or trying to make a fist.

No preoperative imaging studies was done to the patient.

Routine investigations and surgical fitness were done for the patient.

### 2.1 Operative details

The preoperative plan was to reconstruct the FDP tendon by using the FDS tendon as a free tendon graft in the same sitting if the pulleys are intact or to do staged tendon reconstruction in case of damaged pulleys or if there was significant scarring.

Under general anesthesia and tourniquet control, Bruner-type incision for exploration revealed cut of both FDP, FDS tendons zone II of the patient's left Ring finger and both digital nerves.

The distal stumps of FDP and FDS tendon maintained the integrity of A2 and A4 pulleys, after further exploration for A1 pulley, A1, A2 and A4 pulleys were salvaged.

Proximal part of FDP tendon was identified by the origin of the lumbrical muscle but surprisingly there was no free end and the FDS proximal stump was not found.

**Fig. 1:**
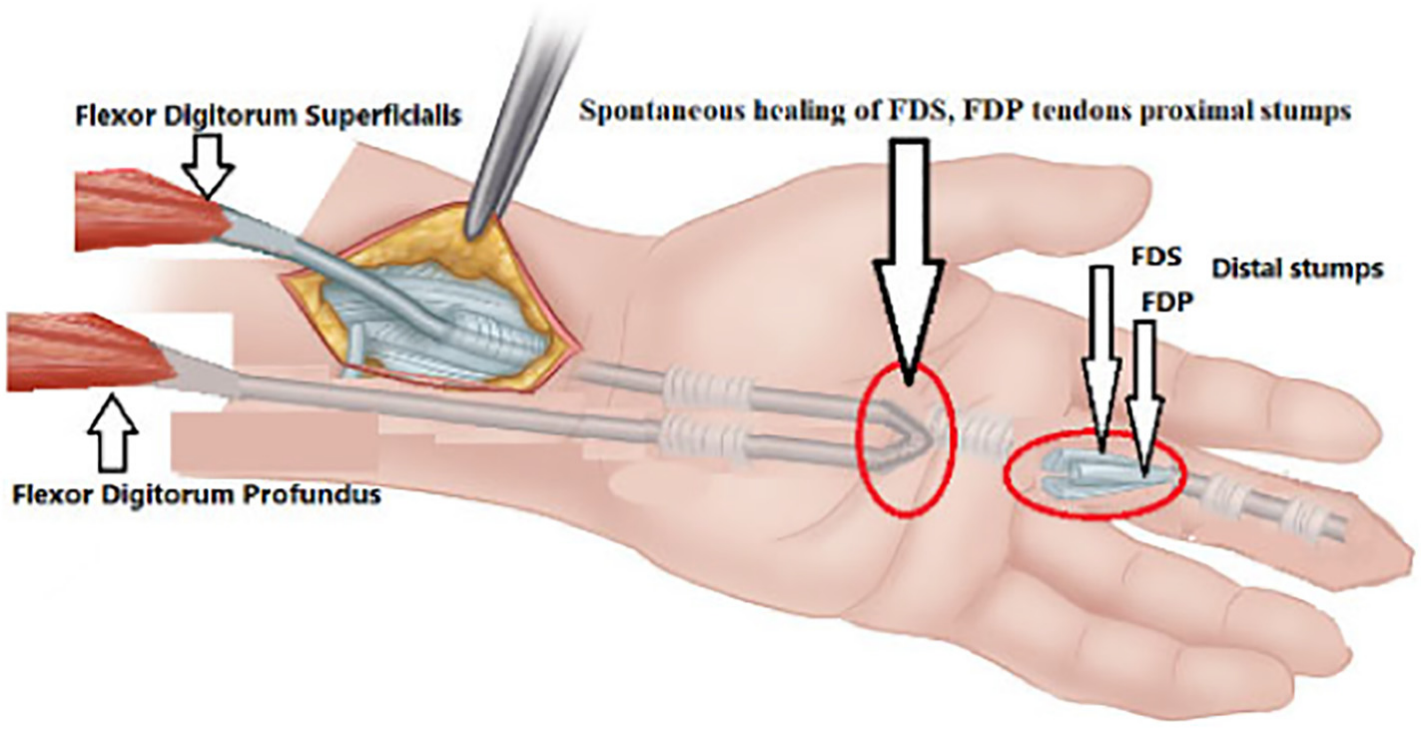

**Fig. 2:**
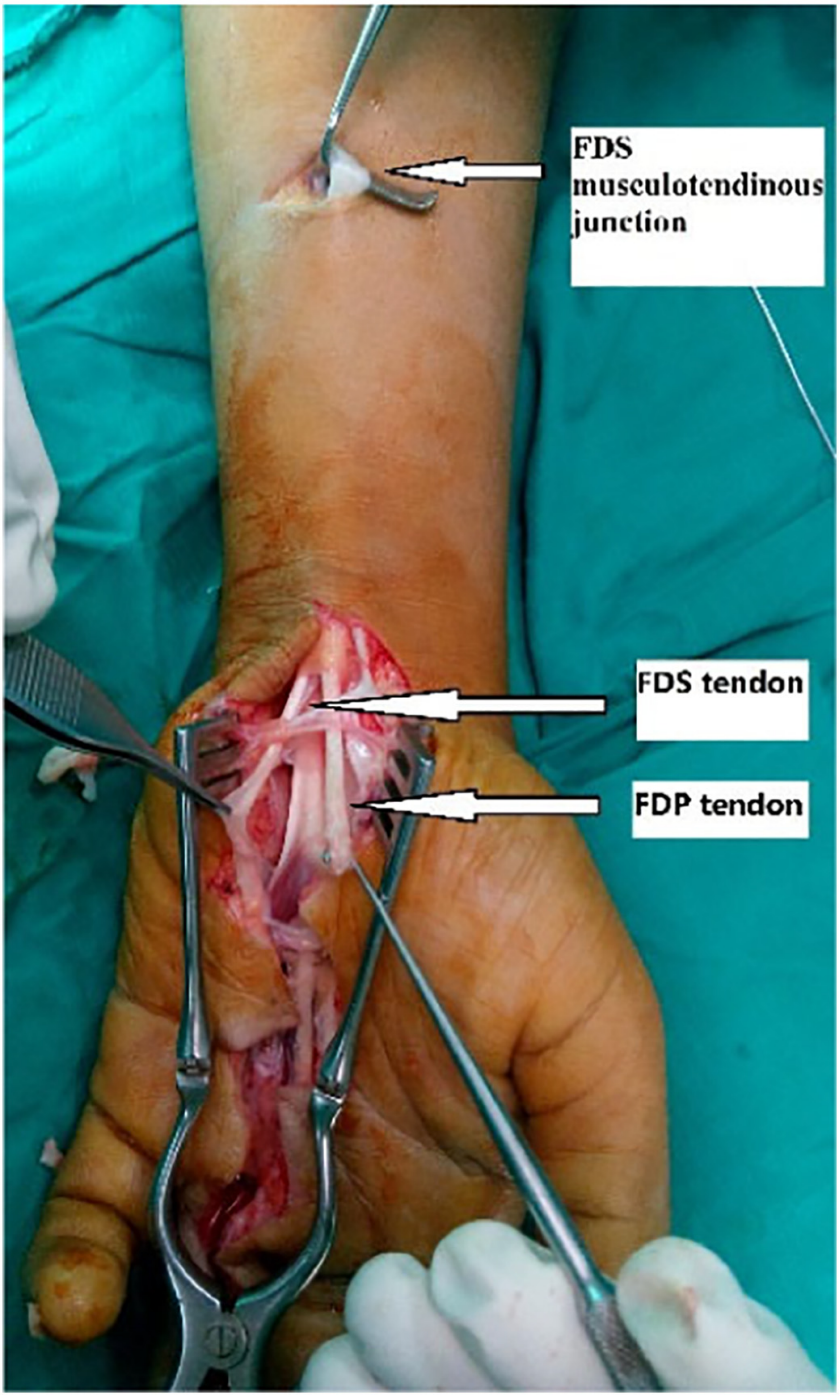

**Fig. 3:**
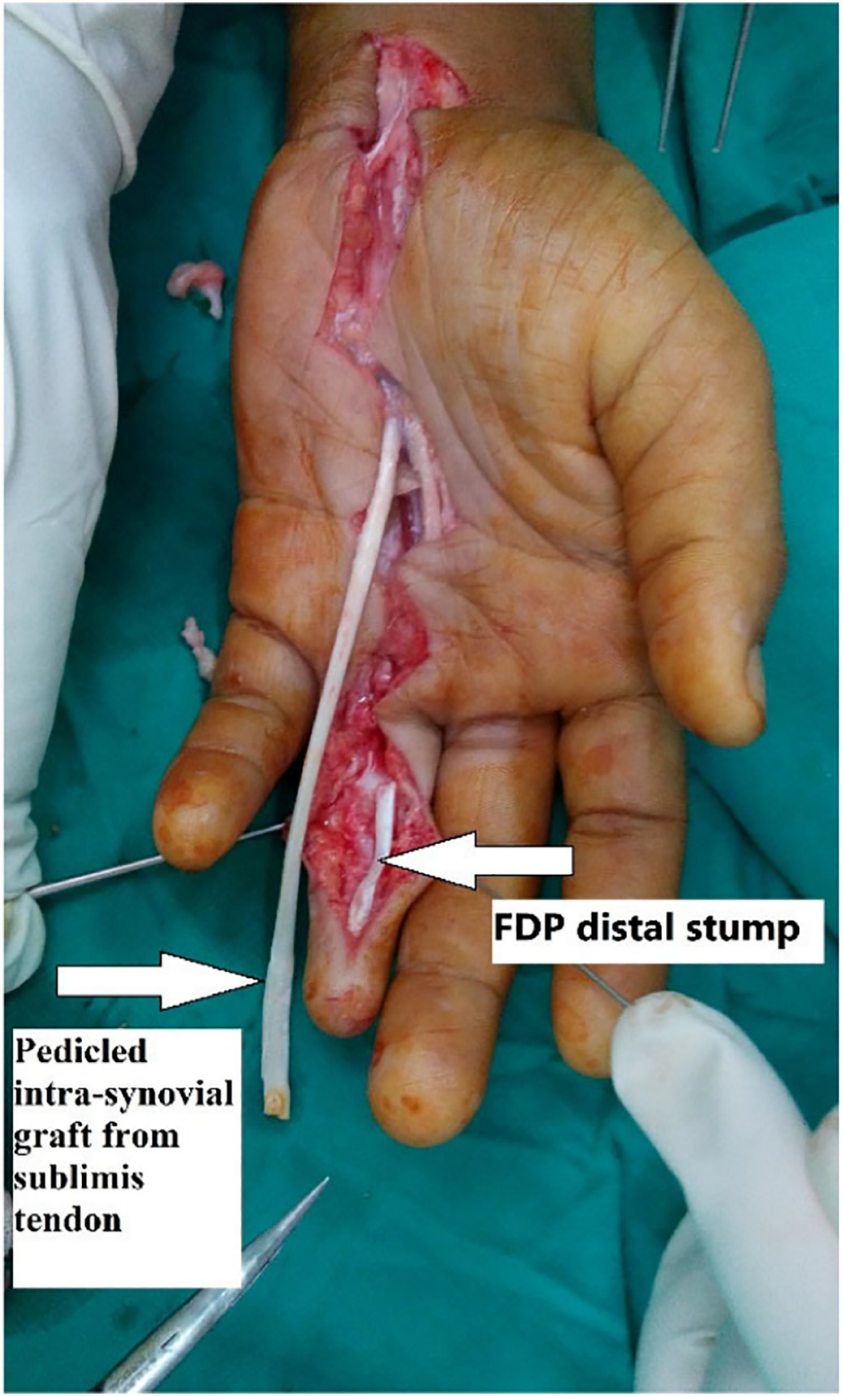

Upon tracing the FDP tendon proximally it was noticed that the two ends of the FDP, FDS tendons were healed spontaneously together forming a loop with the FDS muscle at one end and the FDP at the other end with single tendon in between as shown in the diagram (Fig. 1), this tendon was cut proximally at the musculotendinous junction of FDS tendon to gain more length while the tendon is still attached to the FDP muscle (as pedicled intra-synovial graft from sublimis tendon) (Fig. 2). Pulling on the tendon to retrieve the end to the palm (Fig. 3), then passing the tendon through A1, A2 and A4 pulleys and using the modified Bunnell (tendon-to-bone pull-out) to insert the tendon to the distal phalanx after adjusting the tension and removal of excess length.

**Fig. 4:**
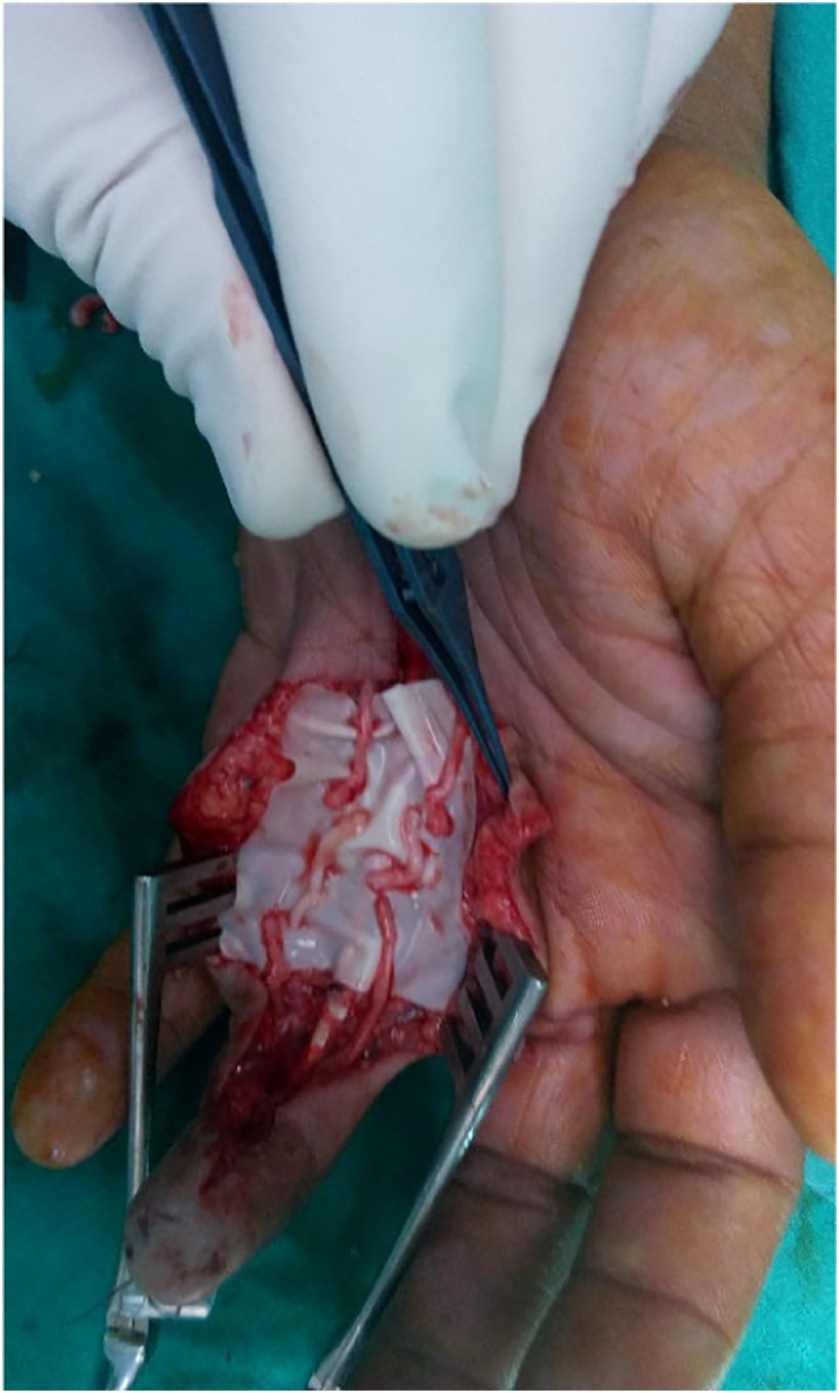

For tension free repair of bilateral digital nerves, free nerve grafts were harvested from ipsilateral medial cutaneous nerve of the forearm to reconstruct the nerve defects which was (2.5 and 3.5 cm) (Fig. 4).

Closure of the skin and application of posterior slab to maintain flexion of the wrist and metacarpophalangeal joints.

### 2.2 Follow-up

Immediate passive flexion of finger was allowed using the patient's non-injured hand.

The patient had regular follow ups at 2 weeks for removal of sutures, at 4 weeks for removal of slab and referral to physiotherapy, at 2 and 4 months.

### 2.3 Results

The patient was followed-up for 4 months and after completion of the physiotherapy program, she regained the ability to actively flex her finger to near full flexion with improved function and cosmesis, range of motion for the index metacarpophalangeal joint was 90, proximal inter-phalangeal was 90, distal inter-phalangeal joint was 50.

## 3 Discussion

Delayed flexor tendon reconstruction in neglected cases is still offering good results even after long periods of delay provided that the finger's joints are still supple and mobile.

Other reports from literature showed good results after flexor tendon reconstruction in neglected cases (e.g. [[9], [10], [11], [12]]).

Pulvertaft [9] reported nine cases where the interval was over three years, the longest being 24 years, Boyes and Stark [10] who had cases of flexor tendon grafting after a delay of three or more years, Miller et al. [11] reported seven patients in whom the interval varied between seven and eighteen years, average thirteen years, furthermore M. W. Jones and J. P. Matthews [12] had reported a case report of tendon grafting after 48 years with good outcome.

Regarding the surgical techniques used for flexor tendon reconstruction in neglected cases E. Paneva-Holevich has modified a technique for staged flexor tendon reconstruction by using pedicled intra-synovial graft from sublimes tendon which offers less incidence of adhesions if compared to the free tendon graft [2,6,7].

In our case report we did one stage surgery using pedicled intra-synovial graft from sublimes tendon after spontaneous healing of the proximal stumps of FDP and FDS tendons.

## Declaration of competing interest

The author has nothing to disclose. No funding was received for this article.

Written informed consent was obtained from the patient for publication of this case report and accompanying images. A copy of the written consent is available for review by the Editor-in-Chief of this journal on request.
